# Angiogenic response of rat hippocampal vasculature to focused ultrasound-mediated increases in blood-brain barrier permeability

**DOI:** 10.1038/s41598-018-30825-8

**Published:** 2018-08-15

**Authors:** Dallan McMahon, Ethan Mah, Kullervo Hynynen

**Affiliations:** 1 Sunnybrook Research Institute, Toronto, M4N 3M5 Canada; 2 University of Toronto, Department of Medical Biophysics, Toronto, M4N 3M5 Canada; 3 University of Toronto, Institute of Biomaterials and Biomedical Engineering, Toronto, M5S 3G9 Canada

## Abstract

Focused ultrasound (FUS) and circulating microbubbles can induce a targeted and transient increase in blood-brain barrier permeability. While preclinical research has demonstrated the utility of FUS for efficacious drug deliver to the brain, there remain gaps in our knowledge regarding the long-term response of brain vasculature to this intervention. Previous work has demonstrated transcriptional changes in hippocampal microvessels following sonication that are indicative of the initiation of angiogenic processes. Moreover, blood vessel growth has been reported in skeletal muscle following application of FUS and microbubbles. The current study demonstrates that blood vessel density in the rat hippocampus is modestly elevated at 7 and 14 d post-FUS compared to the contralateral hemisphere (7 d: 10.9 ± 6.0%, p = 0.02; 14 d: 12.1 ± 3.2%, p < 0.01), but returns to baseline by 21 d (5.9 ± 2.6%, p = 0.12). Concurrently, relative newborn endothelial cell density and frequency of small blood vessel segments were both elevated in the sonicated hippocampus. While further work is required to determine the mechanisms driving these changes, the findings presented here may have relevance to the optimal frequency of repeated treatments.

## Introduction

Focused ultrasound (FUS), in conjunction with circulating microbubbles (MBs), can be used to induce a targeted and transient increase in blood-brain barrier (BBB) permeability^1^, providing a unique approach for the delivery of drugs from the systemic circulation into the brain. A large variety of therapeutic agents have been shown to cross the BBB following sonication^2–10^, with positive therapeutic effects observed^6,7,11–14^. Promising preclinical work demonstrating efficacy and relative safety, as well as a pressing need for new strategies in the treatment of brain diseases, has to lead to the initiation of 3 human trials (ClinicalTrials.gov identifiers: NCT02343991, NCT02986932, NCT03119961). While preclinical research has demonstrated the potential of FUS, there remain gaps in our knowledge regarding the long-term response of brain vasculature. This may have considerable relevance for diseases and treatment strategies that require multiple FUS exposures in short succession.

Previous work has demonstrated significant and enduring blood vessel growth in skeletal muscle following the application of FUS and MBs^15–17^; however, it is important to note that sonication parameters used in these studies differ significantly from what is employed in BBB applications. Nevertheless, in the rat brain, with FUS exposures designed to induce transient increases in BBB permeability, transcriptional changes have been observed in hippocampal microvessels 24 hrs following sonication that are indicative of the initiation of angiogenic processes^18^. These changes in gene expression may be closely related to the increased hippocampal neurogenesis^19,20^, glial cell activation^21–24^, and amyloid beta plaque clearance^21,23–25^ that have also been observed to follow FUS, all of which may be initiated by an acute inflammatory response^18,21–23,26^; however, these links have not been demonstrated experimentally.

Despite the fact that brain vasculature is the specific target of FUS in the context of BBB applications and experiences the largest magnitude of physical stress, blood vessel growth has yet to be studied in detail following sonication. This work aims to explore the impact of increased BBB permeability on vascular density, newborn endothelial cell (EC) density, vascular endothelial growth factor (VEGF) A immunoreactivity, and blood vessel diameter at 7, 14, and 21 d post-FUS. While multi-exposure studies suggest FUS has limited and/or temporary impact on behaviour in healthy animals^25,27,28^, the threshold for a safe treatment repetition frequency has yet to be established; the work presented here may provide insight into this question.

## Materials and Methods

### Animals

Male Sprague Dawley rats (n = 24), weighing 200–300 g on the day of sonication, were used in this study (Taconic Biosciences, Germantown, NY, USA). Animals were housed in the Sunnybrook Research Institute animal facility (Toronto, ON, Canada) and had access to food and water *ad libitum*. All animal procedures were approved by the Animal Care Committee at Sunnybrook Research Institute and are in accordance with the Canadian Council on Animal Care and ARRIVE guidelines.

### Animal Preparation

**Figure 1:**
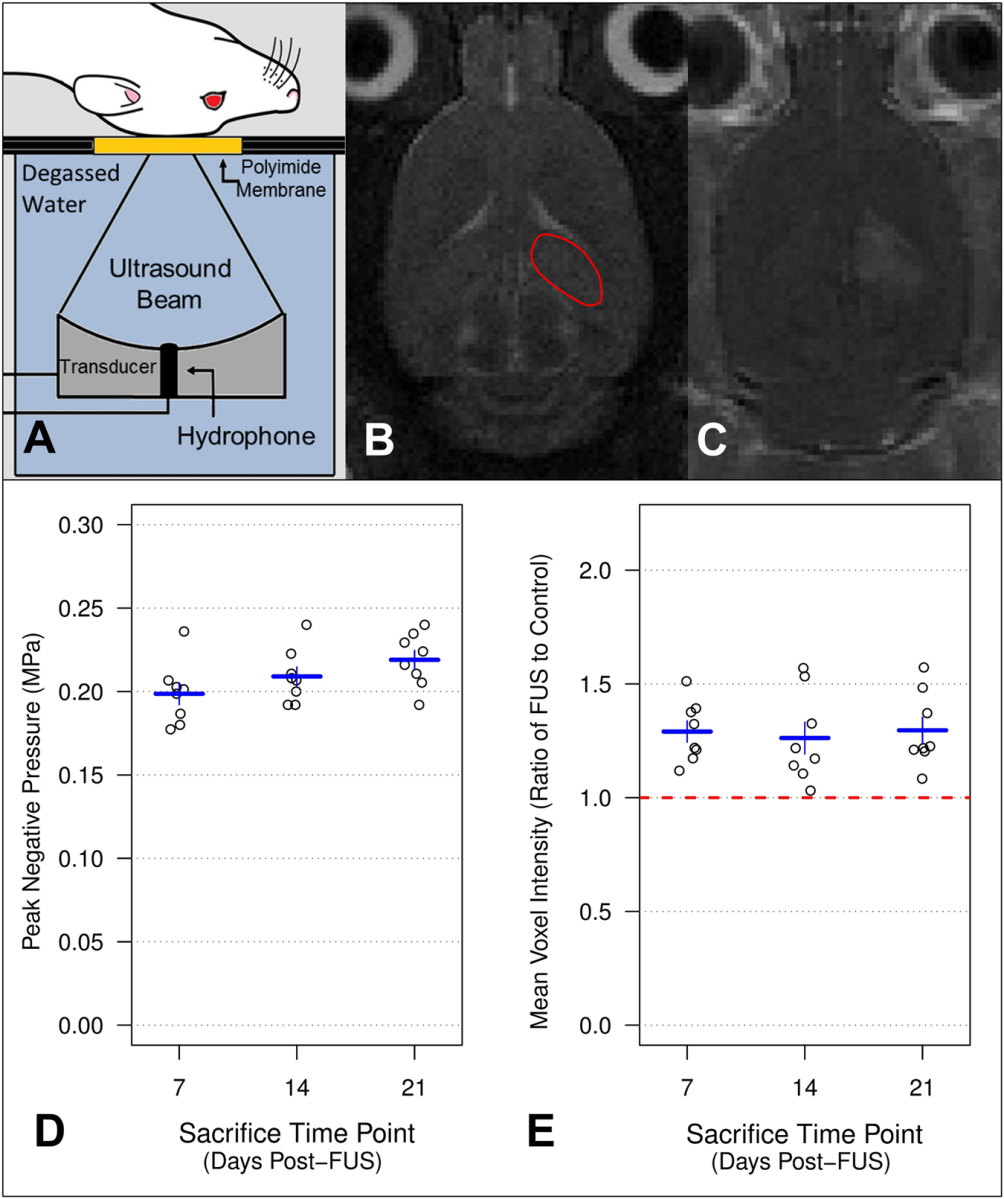
MRIgFUS. During sonication and imaging, rats were positioned supine on an MRI compatible sled with the top of the skull coupled to a polyimide membrane. The bottom of the membrane was coupled to a tank filled with degassed, deionized water, housing the transducer/hydrophone assembly (**A**). The dorsal hippocampus (indicated by red outline) was targeted from T2w images (**B**). Following sonication, contrast enhanced T1w MR images were collected to confirm that BBB permeability was increased (**C**). For rats sacrificed at 7, 14, and 21 d post-FUS, mean PNPs were 199 ± 7 kPa, 209 ± 6 kPa, and 219 ± 6 kPa, respectively (**D**). No significant differences were detected between time points by one-way ANOVA (p = 0.08). When normalized to the contralateral hemisphere, the mean ratio of voxel intensity in the dorsal hippocampus for rats sacrificed at 7, 14, and 21 d post-FUS were 1.29 ± 0.05, 1.26 ± 0.07, and 1.29 ± 0.06, respectively (**E**). No significant differences were detected between time points by one-way ANOVA (p = 0.91). Error bars indicate standard error of the mean. For each sacrifice time point, n = 8.

Anesthesia was induced with 5% isoflurane and oxygen (1 L/min), then maintained at 1.5–2% isoflurane for the duration of the FUS procedure. During sonication and imaging, medical air was used as a carrier gas due to the impact of oxygen on MB circulation half-life^29,30^. Hair overlaying the skull was removed with depilatory cream, a 22-gauge angiocath was placed in the tail vein, and animals were secured in a supine position on an MRI-compatible sled (Fig. 1A). The sled allows movement of the animal between the bore of the MRI and the FUS system. The dorsal surface of the skull was coupled to a polyimide membrane with ultrasound gel and body temperature was maintained with heated saline bags.

### MRIgFUS

Sonications were performed using the LP100 system (FUS Instruments Inc., Toronto, ON, Canada) equipped with a spherically focused transducer (focal number = 0.8, external diameter = 75 mm, internal diameter = 20 mm, frequency = 551.5 kHz), calibrated using a planar fiber optic hydrophone with an active tip diameter of 10 μm (Precision Acoustics Ltd., Dorset, UK). The transducer was situated in a tank of degassed, deionized water and its movement was controlled with a motorized positioning system (3 degrees of freedom). To allow ultrasound propagation from the transducer to the brain, the bottom of the polyimide membrane (part of the sled) was coupled to the water tank below.

Spatial coordinates of the FUS positioning system were coregistered to that of a 7-Tesla MRI scanner (BioSpin 7030, Bruker, Billerica, MA, USA), allowing sonications to be targeted based on axial T2-weighted (T2w; Fig. 1B) images (TR = 2000, TE 60). Three targets in either the right or left dorsal hippocampus were chosen in each animal. Immediately prior to sonication, a bolus of MBs (20 μl/kg; Definity, Lantheus Medical Imaging, North Billerica, MA, USA) diluted in saline, followed by a gadolinium-based contrast agent (0.2 ml/kg; Gadovist, Schering AG, Berlin, Germany) were administered via tail vein catheter.

Ultrasound was delivered in 10 ms bursts with a pulse repetition frequency of 1 Hz for 120 s. Starting peak negative pressure (PNP) was set at 0.128 MPa (measured in water) and increased by an increment of 0.008 MPa each second. During sonication, acoustic emissions were monitored with an in-house manufactured polyvinylidene difluoride hydrophone located in a small perforation in the centre of the transducer. Once the ratio of signal to baseline at the first or second ultraharmonic frequency passed 3.5, the sonicating pressure was dropped by 50% and maintained at this level for the remainder of sonication. This algorithm is designed to calibrate pressure based on *in vivo* MB response, producing a more consistent increase in BBB permeability compared to a set pressure strategy^31^. T1w images (TR = 500, TE 10) were collected approximately 10 minutes following sonication to assess BBB permeability (Fig. 1C).

To label proliferating cells, bromodeoxyuridine (BrdU) was administered daily (50 mg/kg; 10 mg/ml diluted in saline; i.p.), starting 24 hrs post-FUS, until the day before sacrifice.

### Immunohistochemistry

At 7, 14, and 21 d following sonication, rats were transcardially perfused with ice-cold phosphate buffer (PB; 0.1 M, pH 7.4), followed by 4% paraformaldehyde in PB. Brains were extracted, post-fixed for 24 hrs at 4 °C, then transferred to 30% sucrose in PB and stored at 4 °C until sinking to the bottom of the storage vessel (~3 days). Brains were embedded in optimum cutting temperature compound (Tissue-Tek, Torrance, CA, USA) and stored at −80 °C. Coronal cryostat sections (40 μm) were stored in cryoprotectant (glycerin, ethylene glycol, and 0.2 M PB in a ratio of 2:3:5, respectively) at −10 °C until immunohistological processing.

Co-staining for BrdU and glucose transporter 1 (GLUT1) was performed on free-floating sections with sequential primary antibody incubations. Briefly, after sections were washed in PBST, antigen retrieval proceeded with 90 min in 2 M HCl at room temperature followed by a wash, 10 min in 0.1 M borate buffer (pH 8.5), and 2 additional washes. Sections were blocked for 1 hr at room temperature (0.1% Triton X-100, 1% bovine serum albumin, 2% goat serum, 1x PBS), then incubated in rat anti-BrdU primary antibody (1:400; OBT0030, Bio-Rad, Hercules, CA, USA), diluted in blocking buffer, overnight at 4 °C. After 3 washes, sections were incubated in goat anti-rat IgG Alexa Fluor 488 secondary antibody (1:400; ab150165, Abcam Inc, Cambridge, MA, USA) for 2 hrs at 4 °C, washed 3 times, then incubated in rabbit anti-GLUT1 primary antibody (1:400; ab15309, Abcam Inc, Cambridge, MA, USA), diluted in blocking buffer, overnight at 4 °C. After 3 washes, sections were incubated in goat anti-rabbit IgG Alexa Fluor 647 secondary antibody (1:400; ab150079, Abcam Inc, Cambridge, MA, USA) for 2 hrs at 4 °C and mounted onto X-tra glass slides (Leica Microsystems, Wetzlar, Germany) with aqueous mounting media (Fluoroshield™ with DAPI, Sigma-Aldrich Corporation, St. Louis, MO, USA). Slides were stored in the dark at 4 °C until imaging.

In another series of sections, immunohistochemical staining proceeded as indicated above, however, mouse anti-VEGFA primary antibody (1:400; ab1316, Abcam Inc, Cambridge, MA, USA) was used in place of the BrdU primary antibody and rabbit anti-mouse Alexa Fluor 555 (1:400; ab150126, Abcam Inc, Cambridge, MA, USA) was used as secondary antibody.

### Confocal Imaging

For quantification of blood vessel density, blood vessel diameter, and newborn EC density, image stacks (2 μm spacing, 1.24 μm/pixel, 512 × 512-pixel field of view) were collected through the entire section thickness, bilaterally, in each of the 3 major subfields of the hippocampus, cornus amonis 1 (CA1), CA3, and dentate gyrus (DG). Sections (7–9 per animal) were imaged with a 20x objective (NA 0.75) using a confocal laser microscope system (A1+, Nikon, Tokyo, Japan) at excitation/emission wavelengths of 639.1 nm/700.0 nm, 488.0 nm/525.0 nm, and 403.1 nm/450.0 nm for GLUT1, BrdU, and DAPI, respectively. To ensure subsequent image analysis was performed on equivalent regions of the hippocampus, z-stacks were rotated and cropped to include only regions of CA1 and CA3 between stratum radiatum and stratum oriens, inclusive, as well as only regions of DG between the granular cell layer and the outer molecular layer. Image stacks were also cropped in the z-axis, keeping 5 images starting at the first complete optical section.

### Blood Vessel Density

**Figure 2:**
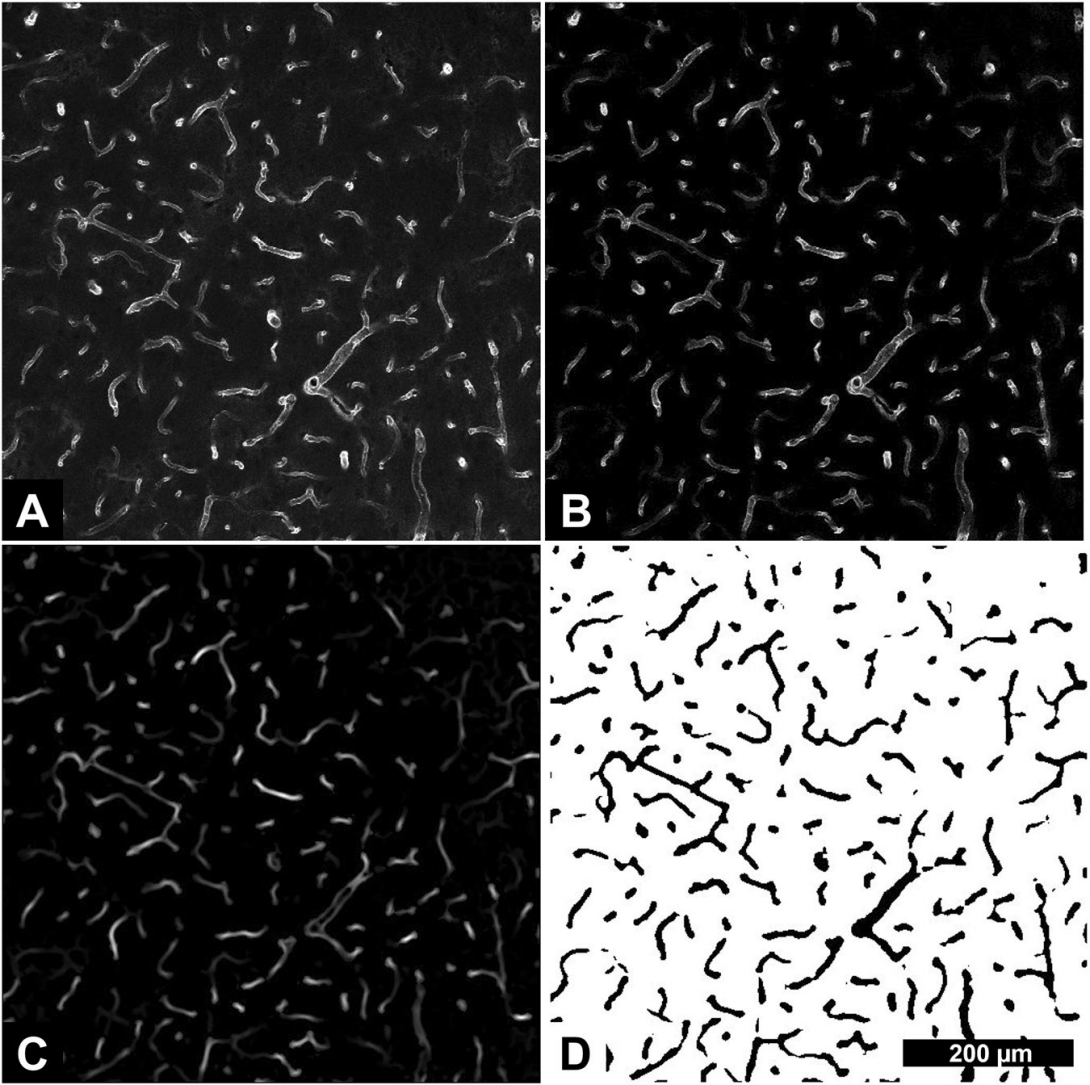
Blood vessel segmentation pipeline. Image stacks were collected for the full thickness of sections (2 μm spacing, 1.24 μm/pixel, 512 × 512-pixel field of view). Maximum intensity projections (5 images after the first complete optical slice) of the GLUT1 channel (**A**) were used to assess blood vessel density. Subtraction of the BrdU channel, with high intensity regions (BrdU positive cells) thresholded out, was used to reduce background intensity (**B**). ImageJ macro, *Tubeness*, was used to identify “tube-like” structures (**C**). The outputs of *Tubeness* were thresholded and masked (**D**). Binary mask images were normalized to imaging volume to determine blood vessel density. All image segmentation steps were performed in ImageJ. For each sacrifice time point, n = 8.

GLUT1 positive blood vessels (Fig. 2A) were segmented using an in-house designed ImageJ pipeline. First, background signal intensity in the GLUT1 channel was reduced by subtraction of the BrdU channel with high intensity pixels (BrdU positive cells) removed with global thresholding (Fig. 2B). Next, blood vessels were segmented from maximum intensity projections with an ImageJ macro, *Tubeness* (Fig. 2C). Tube-like structures were extracted by thresholding and binary masking of *Tubeness* outputs (Fig. 2D). GLUT1 positive area (using binary mask images) was normalized to imaging volume for each image stack and averaged within animals for each subfield. Density ratios (sonicated to control hemisphere) for each subfield were log2 transformed and averaged within animals to obtain hippocampal means.

### Blood Vessel Diameter

Maximum intensity projections of cropped GLUT1 channel z-stacks were used to quantify the diameter of blood vessel segments. Using ImageJ plugin, *ObjectJ*, the diameter of each blood vessel segment was manually measured by an author (DM) blinded to treatment. Diameter histograms, binned to < 5 μm, 5–7.5 μm, 7.5–10 μm, and > 10 μm (integer multiples of the spatial resolution of images), were normalized to image stack volumes. Density ratios (sonicated to control hemisphere) were calculated between hemispheres and log2 transformed.

### Newborn EC Density

**Figure 3:**
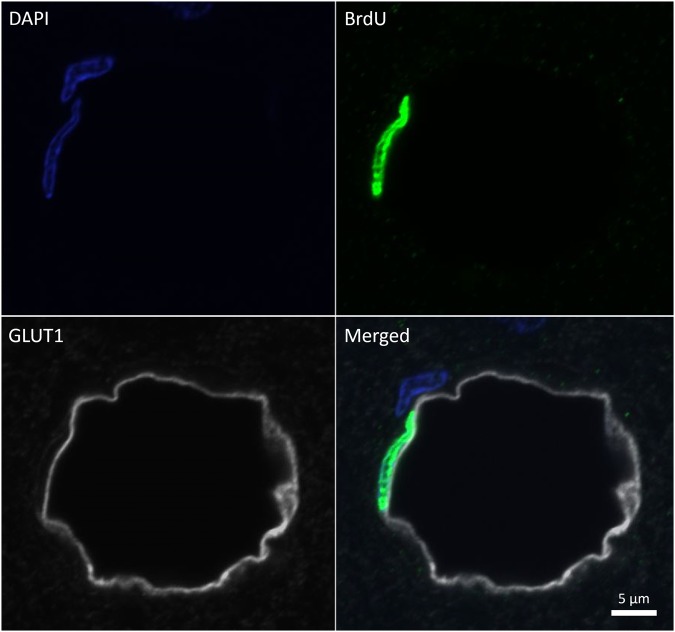
BrdU positive endothelial cell example. Newborn ECs were defined as the colocalization of BrdU, GLUT1, and DAPI with a nuclei shape characteristic of ECs: elliptical in shape with major axes aligned to the long axes of blood vessels. Newborn ECs were found lining the full spectrum of blood vessel sizes in the hippocampus. For each sacrifice time point, n = 8.

Cropped image stacks (GLUT1, BrdU, and DAPI channels) were used to quantify the density of newborn ECs. An author (EM) blinded to treatment manually quantified the number of BrdU positive ECs, defined as the colocalization of BrdU, GLUT1, and DAPI with a nuclei shape characteristic of ECs (oblong/elliptical in shape with major axes aligned to the long axes of blood vessels; Fig. 3). Newborn EC density was normalized to imaging volume for each image stack and averaged within animals for each subfield. Density ratios (sonicated to control hemisphere) for each subfield were log2 transformed and averaged within animals to obtain hippocampal means. In some regions of brain tissue, traces of BrdU+ blood cells were evident in blood vessels due to poor perfusion; these subfields/sections/animals were not included in subsequent analyses.

### Statistics

All statistical analyses were performed in R 3.4.3. Repeated measures, two-way, analysis of variance (ANOVA) was performed at each time point for blood vessel density, blood vessel diameter, and newborn EC density with subfield specific post-hoc student’s t-tests (two-sample, two-tailed, paired). FDR correction for multiple comparisons was used to account for the analysis of 3 time points and 3 subfields. Contrast-enhancement and PNP were compared between animals at different time points with one-way ANOVA. For all analyses, an alpha of 0.05 was used as the threshold for statistical significance.

## Results

### Equivalent PNP and contrast enhancement across time points

During sonication, acoustic emissions were monitored. Once the ratio of signal to baseline at the first or second ultraharmonic frequency passed 3.5 (threshold event), the PNP was dropped by 50% and maintained at this level for the remainder of sonication. PNP after detecting a threshold event was not significantly different between groups (Fig. 1D). For rats sacrificed at 7, 14, and 21 d post-FUS, mean PNPs were 199 ± 7 kPa, 209 ± 6 kPa, and 219 ± 6 kPa, respectively (p = 0.08; one-way ANOVA).

Ten minutes following sonication, T1w images were collected to confirm FUS-mediated increases in BBB permeability and to quantify relative contrast-enhanced signal intensity. No significant differences were observed between groups (Fig. 1E). When normalized to the contralateral hemisphere, mean relative voxel intensities in the sonicated dorsal hippocampus for rats sacrificed at 7, 14, and 21 d post-FUS were 1.29 ± 0.05, 1.26 ± 0.07, and 1.29 ± 0.06, respectively (p = 0.91; one-way ANOVA).

### FUS induces an increase in the density of newborn ECs

**Figure 4:**
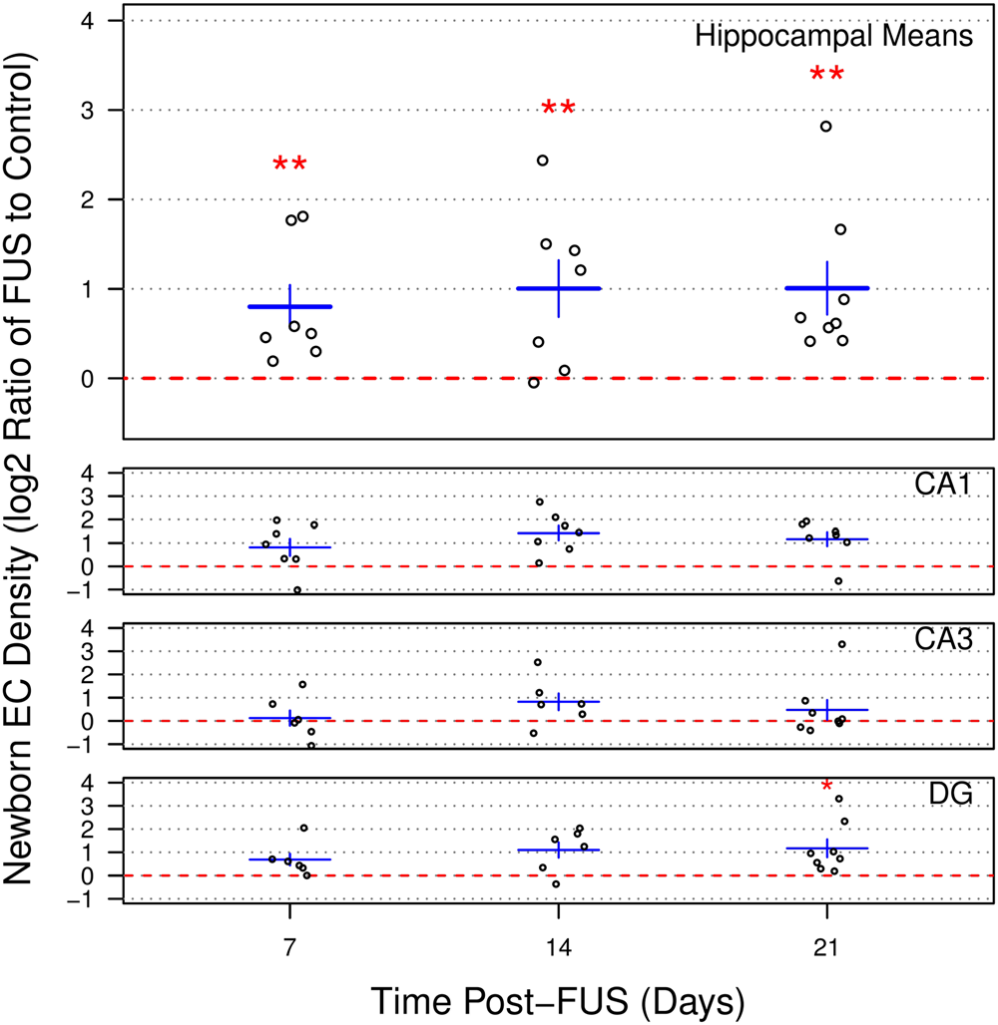
Newborn endothelial cell density following sonication. Newborn ECs density in the sonicated hippocampus was significantly increased at all time points relative to the contralateral hemisphere. Density ratios (relative to control hemisphere; log2 scale), averaged across the hippocampus, of 0.785 ± 0.242, 1.003 ± 0.317, and 1.006 ± 0.295 were measured at 7, 14, and 21 d post-FUS, respectively. These log2 ratios correspond to increases in newborn EC density in the sonicated hemispheres of 72.4 ± 18.3%, 100.4 ± 24.6%, and 100.9 ± 22.7%, respectively. Post-hoc analysis at each time point revealed significantly increased newborn EC density in the sonicated hemisphere 21 d post-FUS in DG (log2 ratio of 1.173 ± 0.385, FUS to control). No significant differences were detected between time points. Red dotted lines at y = 0 indicate no difference between sonicated and control hemispheres. * indicates p < 0.05. ** indicates p < 0.01. Error bars represent standard error of the mean. n = 7, 7, and 8 for sacrifice time points of 7, 14, and 21 d post-FUS, respectively (poorly perfused sections were eliminated from analyses).

Newborn EC density in the sonicated hippocampus was significantly increased at all time points relative to the contralateral hemisphere. Relative increases of 72.4 ± 18.3%, 100.4 ± 24.6%, and 100.9 ± 22.7% (p < 0.01 for all time points) were observed in the sonicated hippocampus at 7, 14, and 21 d post-FUS, respectively (Fig. 4). Post-hoc analysis at each time point revealed significantly increased newborn EC density in the sonicated hemisphere 21 d post-FUS in DG (p = 0.02; 125.5 ± 30.6%); however, post-hoc analyses may be underpowered for the detection significant differences when accounting for multiple comparisons. No significant differences were detected between time points.

Qualitative evaluation of newborn ECs following FUS revealed no obvious pattern with regards to the size of the blood vessels that they lined; BrdU positive ECs were found lining the full spectrum of blood vessel sizes in the hippocampus.

### Blood vessel density is increased at 7 and 14 d following FUS

**Figure 5:**
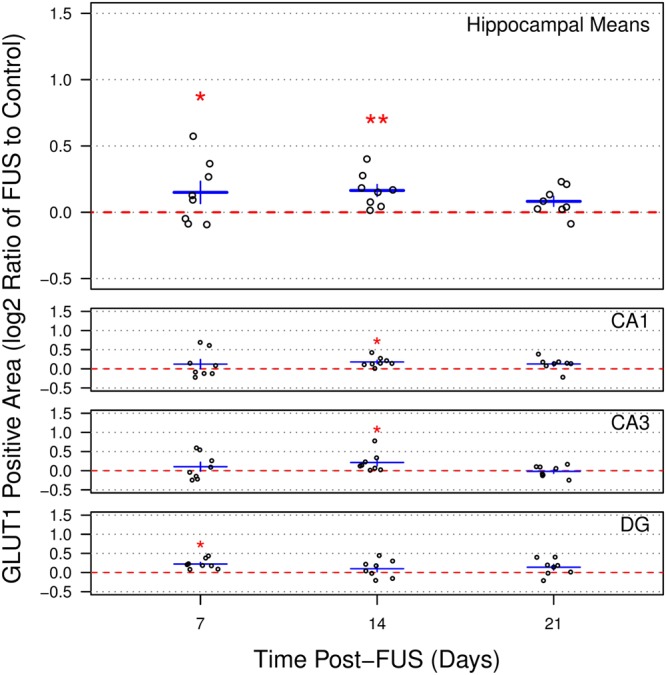
Hippocampal blood vessel density following sonication. Relative blood vessel density was assessed by log2 ratio of GLUT1 immunoreactive area (normalized to imaging volume) in the sonicated vs non-sonicated hemispheres. At 7 and 14 d post-FUS, mean log2 density ratios across the hippocampus of 0.149 ± 0.084 and 0.164 ± 0.045, respectively, were measured. These log2 ratios correspond to increases in blood vessel density in the sonicated hemispheres of 10.9 ± 6.0% and 12.1 ± 3.2%, respectively. No significant differences were observed across the hippocampus 21 d following sonication (log2 ratio of 0.083 ± 0.037, p = 0.12). Post-hoc analysis at each time point revealed significantly increased mean log2 density ratios in DG at 7 d post-FUS (0.229 ± 0.044), as well as in CA1 and CA3 at 14 d post-FUS (CA1: 0.180 ± 0.044; CA3: 0.213 ± 0.089). No significant differences were detected between time points. Red dotted lines at y = 0 indicate no difference between sonicated and control hemispheres. * indicates p < 0.05. ** indicates p < 0.01. Error bars represent standard error of the mean. For each sacrifice time point, n = 8.

**Figure 6:**
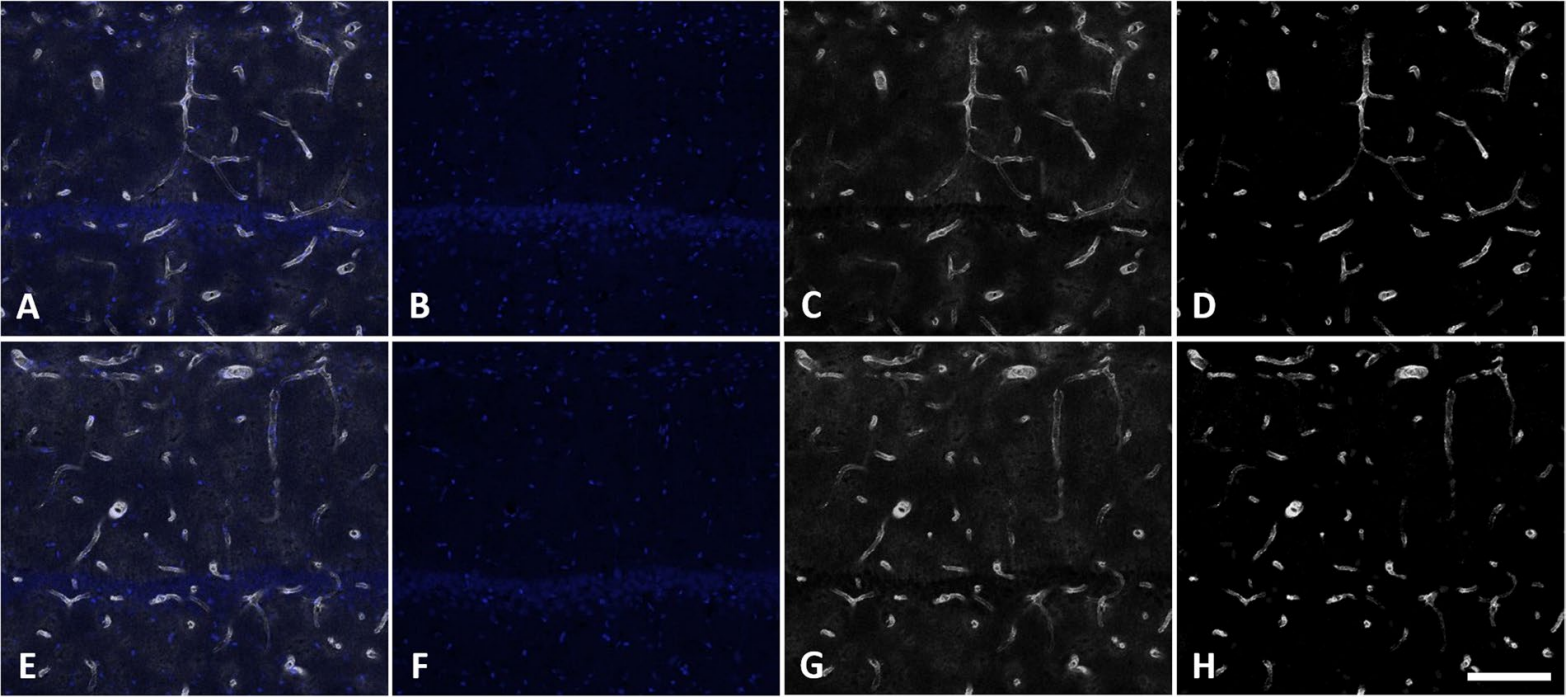
Representative images of increased relative blood vessel density. Images collected in CA1 of the non-sonicated (**A**–**D**) and sonicated (**E**–**H**) hemispheres at 14 d post-FUS demonstrate a small relative increase (14.1%) in GLUT1 immunoreactive area of the sonicated hemisphere. DAPI staining (**B** and **F**), GLUT1 immunoreactivity (**C**,**G**), merged channels (**A**,**E**), as well as GLUT1 immunoreactivity with background subtraction (**D** and **H**), are displayed. Scale bar = 100 μm.

Relative blood vessel density in maximum intensity projections, as assessed by the ratio of GLUT1 immunoreactive area (normalized to imaging volume) in the sonicated vs control hemispheres, was increased across the hippocampus at 7 and 14 d post-FUS (Fig. 5; 7 d: 10.9 ± 6.0%, p = 0.017; 14 d: 12.1 ± 3.2%, p < 0.01). No significant differences were observed across the hippocampus 21 d following sonication (5.9 ± 2.6%, p = 0.12). Post-hoc analysis at each time point revealed significantly increased relative blood vessel density in DG at 7 d post-FUS (16.7 ± 3.1%, p < 0.01), as well as in CA1 and CA3 at 14 d post-FUS (CA1: 13.3 ± 3.1%, p < 0.01; CA3: 15.9 ± 6.4%, p = 0.04). Representative images of increased relative blood vessel density in CA1 14 d post-FUS are displayed in Fig. 6. No significant differences were detected between time points.

### Frequency of small blood vessel segments is increased at 14 d post-sonication

**Figure 7:**
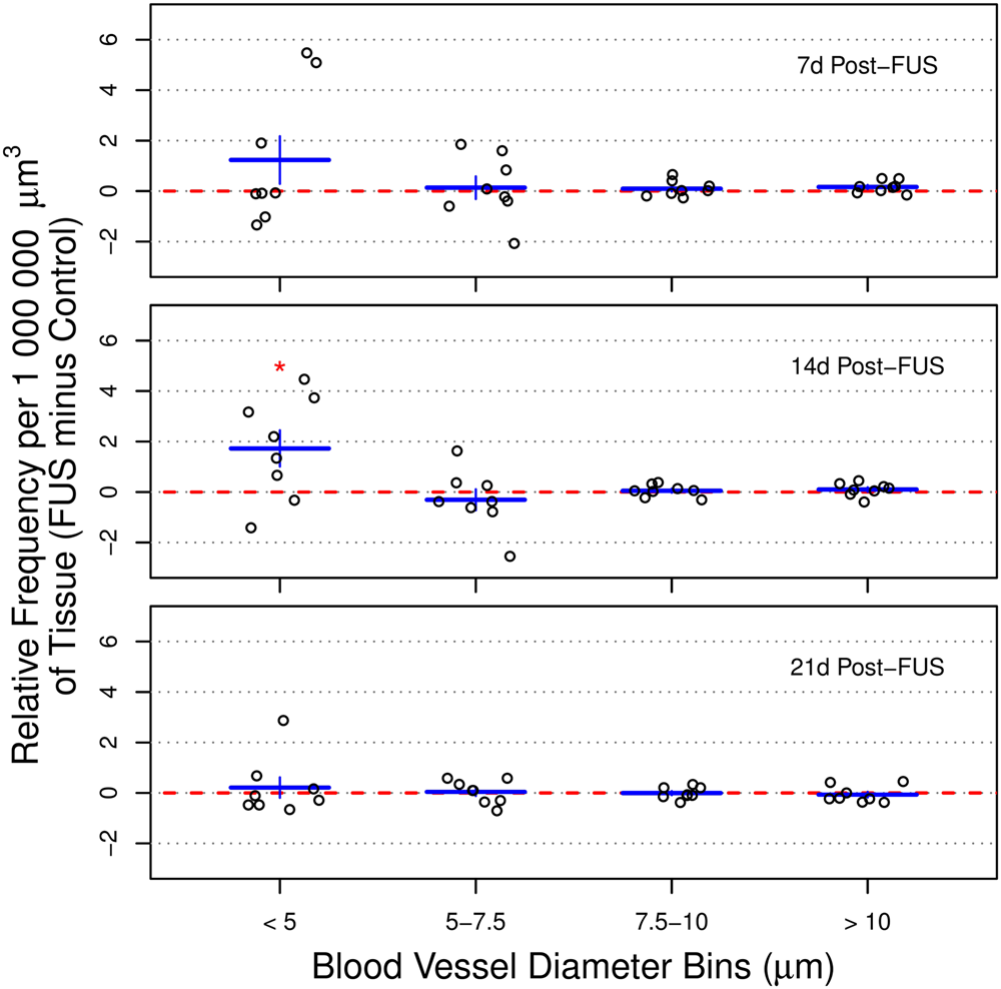
Frequency of blood vessel segment diameters following sonication. Blood vessel segment diameters were compared across the hippocampus in both sonicated and control hemispheres. At 14 d post-FUS, the frequency of small blood vessel segments (<5 μm) was significantly greater in the sonicated hippocampus (1.73 ± 0.72 more small blood vessels in 1 000 000 μm^3^ of brain tissue than control hemisphere). No significant differences were observed at any other time point or for any other bin. At 21 d following sonication, the magnitude of effect size and variance in both the <5 μm and 5–7.5 μm bins was smaller than at 7 or 14 d. Red dotted lines at y = 0 indicate no difference between sonicated and control hemispheres. * indicates p < 0.05. Error bars represent standard error of the mean. For each sacrifice time point, n = 8.

Manually measured, binned, blood vessel segment diameters were compared across the hippocampus in both sonicated and control hemispheres (Fig. 7). At 14 d post-FUS, the frequency of small blood vessel segments (<5 μm) was significantly greater in the sonicated hippocampus (1.73 ± 0.72 more small blood vessels in 1 000 000 μm^3^ of brain tissue than control hemisphere; p = 0.05). No significant differences were observed at any other time point or for any other bin. At 21 d following sonication, the magnitude of effect size and variance in both the <5 μm and 5–7.5 μm bins was smaller than at 7 or 14 d.

### VEGFA immunoreactivity is evident 7 d post-FUS

**Figure 8:**
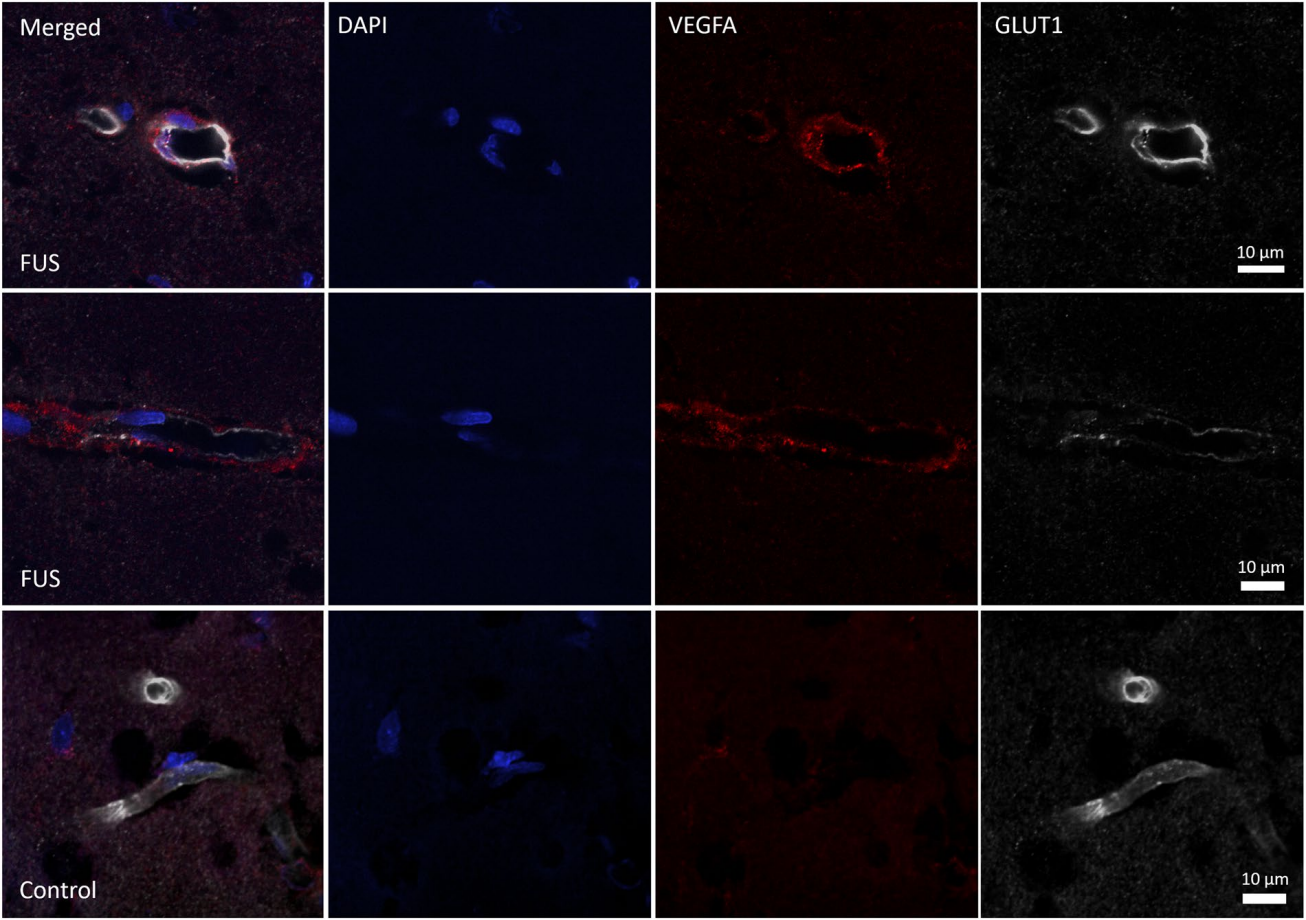
Representative images of VEGFA immunoreactivity 7 d post-FUS. VEGFA immunoreactivity was qualitatively evaluated in sections stained for VEGFA, GLUT1, and DAPI. At 7 d post-FUS, sparse vascular (upper panels) and perivascular (middle panels) VEGFA immunodetection was evident in the sonicated hemisphere for a subset of animals. In the contralateral hemisphere of the same rat (bottom panels), immunodetection of VEGFA was greatly reduced. For each sacrifice time point, n = 8.

VEGFA immunoreactivity was qualitatively evaluated in sections stained for VEGFA, GLUT1, and DAPI. At 7 d post-FUS, sparse vascular and perivascular VEGFA immunodetection was evident in the sonicated hemisphere (Fig. 8) for 4 of 8 animals; minimal levels of VEGFA staining were seen in the contralateral hemisphere. Qualitatively, no differences in VEGFA immunoreactivity were apparent between the sonicated and control hemispheres at 14 or 21 d post-FUS.

## Discussion

Results presented here indicate that a FUS-mediated increase in BBB permeability is followed by a transient, modest increase in hippocampal blood vessel density, accompanied by increases in newborn EC density, the frequency of small blood vessel segments, and VEGFA immunoreactivity. The differences detected 7 and 14 d following sonication appear to normalize to levels found in the contralateral hemisphere by 21 d. To the best of our knowledge, this is the first report of vascular growth following FUS + MBs in brain tissue. Although the mechanisms driving these changes were not thoroughly investigated here, previous work demonstrating an acute inflammatory response following FUS-mediated increases in BBB permeability^18,21–23,26^ may suggest this to be a contributing factor.

While increases in the relative area of GLUT1 immunodetection and BrdU positive EC density indicate blood vessel growth in general, an increase in the frequency of blood vessel segments smaller than 5 μm in diameter may suggest that sprouting angiogenesis is responsible for these morphological changes; angiogenesis proceeds with the breakdown of basement membranes and sprouting of new blood vessels from existing vasculature, followed by a maturation of size and function. Previous work has shown that gene expression for proteins involved in basement membrane breakdown, such as matrix metalloproteinase 9^32^ and cathepsin B^33^, as well as other proteins implicated in EC proliferation, including galectin 3^34^ and early growth response 3^35^, are upregulated following FUS^18^. Thus, an increase in the relative frequency of small blood vessel segments 14 d following sonication, combined with previously characterized gene expression changes, may suggest that new blood vessels are being formed following sonication through angiogenic processes.

In addition, the observation that BrdU positive ECs were present in both small capillaries and larger vessels in the hippocampus may suggest that circulating endothelial progenitor cells are incorporated into vasculature following sonication. These cells are produced in bone marrow and respond to many of the same chemokines and growth factors that drive angiogenesis^36,37^. The incorporation of circulating endothelial progenitor cells into vasculature may be involved in blood vessel growth and repair following FUS and may contribute to increases in the relative area of GLUT1 immunodetection.

Qualitative analysis of VEGFA immunoreactivity may implicate this signaling pathway as a contributing factor, driving the observed morphological changes. In a subset of rats, sparse vascular and perivascular VEGFA immunodetection was observed in the sonicated hippocampus 7 d after FUS (Fig. 8). Given its well-established role in vascular growth (reviewed in^38^), it is reasonable to hypothesize that increased VEGFA expression following FUS could contribute to the changes in vascular density observed here. Previous work has demonstrated an ~200% increase in VEGF expression 5 days after ultrasound exposure (with Definity MBs) in skeletal muscle, which is accompanied by vascular growth^17^. While the mechanical index (MI = PNP/√frequency) used in this work (MI = 0.7) was substantially higher than that employed in the present study (MI = 0.24–0.32; differences in duty cycle and sonication duration were also present), it is possible that FUS exposures at lower MIs may induce VEGF expression at lower levels. Future work should utilize more quantitative assays to assess this effect in the context of FUS + MBs in the brain. Additionally, the investigation of more acute time points may be necessary to observe protein expression changes that drive these morphological changes.

Increased expression of several growth factors, including fibroblast growth factor (FGF), brain derived growth factor (BDNF), and VEGF, have previously been demonstrated in the acute stages following FUS + MBs in the brain^22^. While these changes may be important drivers in the growth of blood vessels following sonication, it is important to note that there are several key differences in the FUS parameters used in these two studies^39,40^, which make drawing direct comparisons difficult. However, elevated levels of phosphorylated Akt, a downstream signalling molecule of VEGF, at 1.5 and 24 hrs following sonication^41^ with parameters close to those employed in the current study, support the idea that VEGF levels may be elevated in the acute stages following FUS.

Changes in the expression of other well-established drivers of angiogenesis have previously been observed to follow FUS + MBs^18^. Of key importance may be CCL2, a cytokine involved in recruiting monocytes, memory T cells, and dendritic cells to sites of inflammation. Gene expression of Ccl2 is increased at 6 hrs (log2 fold change of 4.73) and its receptor, Ccr2, at 24 hrs (log2 fold change of 4.11) post-FUS^18^. Importantly, CCL2 has previously been shown to induce angiogenesis directly^42^ and indirectly through recruitment of macrophages^43–45^ and subsequent VEGFA production^44^. Other chemokines and cytokines produced in response to FUS, such as IL1β, CCL3, and CXCL1^18^, have also been shown to promote angiogenesis in other contexts^46–49^.

Previous work has demonstrated that an acute inflammatory response, as measured by NfκB pathway activation^22,26^ and the expression of genes related to inflammation^18^, follows FUS + MBs in the brain. Downstream indicators of inflammation and those potentially related to inflammation have also been observed, including glial cell activation^21,23^, neurogenesis^19,20,50,51^, amyloid beta plaque clearance^21,23,25,52^, downregulation of transporters^18,53,54^, and now angiogenesis. The magnitude and implications of these responses are currently the topic of debate^22,26,55,56^. A large body of evidence suggests that single exposures to FUS-mediated increases in BBB permeability result in minimal short term and no long-term behavioral deficits^57–60^. Repeated FUS exposures have similarly been shown to have limited detrimental effects on behavior^25,27,28,60^, and even improvements in mouse models of Alzheimer’s disease^23,25^; however, determining the impact of different treatment repetition frequencies has not been an area of thorough investigation. Results presented here would suggest that 21 days between sonications may serve as a conservative guideline to ensure brain tissue is allowed to fully recover from intervention, reducing the potential for detrimental impacts to accumulate. When considering treatment strategies and disorders for which the risks of minor tissue damage or acute inflammation are less of a concern, such as brain tumors, a less conservative treatment repetition frequency may be warranted.

In the present study, no significant correlations were found between post-FUS BBB permeability and blood vessel density or newborn EC density. T1w MR imaging was used to assess BBB permeability following sonication and while this approach is valuable for confirming increases in BBB permeability, MR methods exist that can provide more quantitative data for assessing FUS treatments. The information provided by dynamic contrast enhanced-MRI, for example, would enable a more accurate assessment of the effects of FUS on BBB permeability and potentially reveal correlations to the bioeffects observed. Alternatively, the angiogenic response following sonication may not follow a linear progression; sampling at course time intervals following FUS may capture blood vessel growth/angiolysis at different phases depending on the magnitude of initial BBB permeability changes.

Another limitation of this work is that it does not differentiate between BrdU positive ECs incorporated into vessels or formed by division and those in which DNA damage was significant. It is important to note that BrdU is incorporated into any cell in which DNA synthesis is occurring, thus it is possible that a portion of the BrdU positive ECs quantified in this work were in the process of DNA damage repair while BrdU was present. Administration of BrdU was delayed until 24 hrs after sonication to reduce the potential for this to significantly impact results.

Lastly, assessment of VEGFA expression following FUS was qualitative. While differences in immunoreactivity between sonicated and control hemispheres were visually apparent in a subset of rats at 7 d post-FUS, the magnitude of this difference was not quantified. Investigation into the time course of VEGFA expression with more quantitative assays is warranted and would provide valuable information as to the magnitude and duration of this response. In addition, this would allow direct comparisons to other conditions in which VEGFA expression is acutely elevated and blood vessel growth results.

Work presented here indicates that a FUS-mediated increase in BBB permeability is followed by a temporary increase in hippocampal blood vessel density, accompanied by increases in newborn EC density, frequency of small blood vessel segments, and sparse VEGF immunoreactivity. Importantly, the differences detected 7 and 14 d following sonication appear to normalize to levels found in the contralateral hemisphere by 21 d. It is possible that an acute inflammatory response following sonication drives the transient morphological changes observed here. These findings may have little significance for the safety of single FUS treatments, given the magnitude of effect and results of previous behavioural studies, but may have relevance to the optimal frequency of repeated treatments. While FUS as a method to increase BBB permeability remains a promising strategy for drug delivery to the brain, this work emphasizes the importance of continuing to characterize the biological changes that follow an increase in BBB permeability in order to tailor treatment strategies to specific disorders and to fully understand the risks of this technique.
